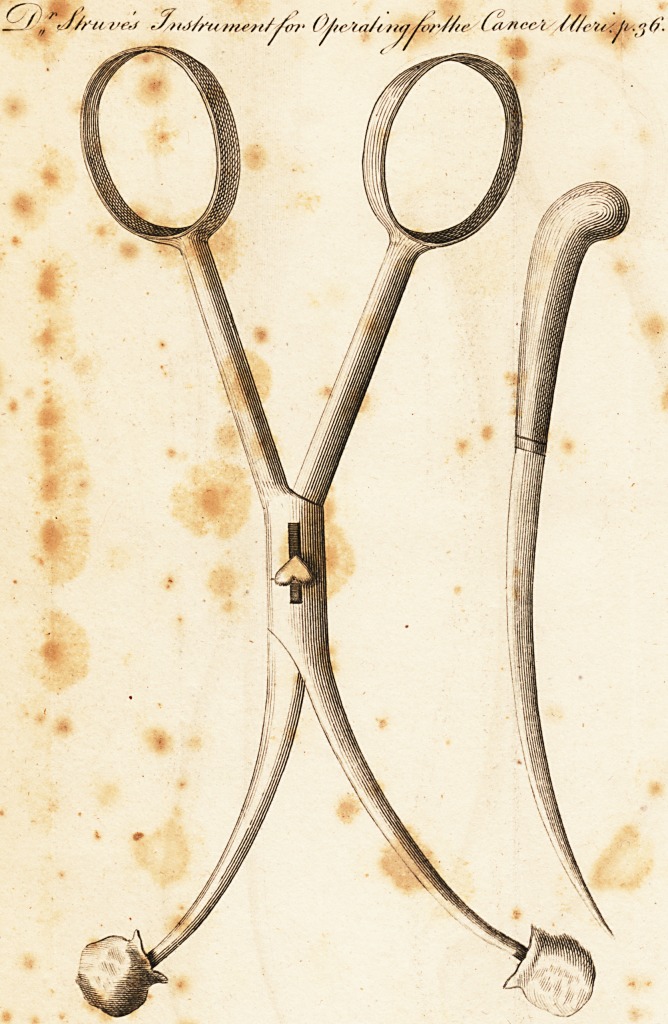# Some Remarks on the Extirpatio Uteri, as Perhaps the Only Method of Operating for the Cancer Uteri

**Published:** 1804-01-01

**Authors:** Ch. Struve


### *Some* Remarks *on the* Extirpatio Uteri, *as perhaps the only method of operating for the* Cancer Uteri, *by Dr.* Ch. Struve.

[ With an Engraving. ]

THE cancer uteri is one of the most dreadful diseases to which the female sex is exposed, and which is generally thought to frustrate the assistance of the healing art so much, that no other consolation can be given to the patient, but what religion affords her, and the hope of death, to put an end to the most insupportable pains with which the disease is attended. However, we find some remedies proposed against this disorder, and even instances are recorded of patients being cured by the internal use of different different remedies; but whatever credit such cases may claim, I have not been so fortunate in my own practice; and in three cases of cancer uteri, that fell under my observation, I have been entirely disappointed in my hopes of cure by all possible remedies, rational as well as empirical. From this melancholy experience I was led to think, whether no help could be afforded from the operative surgery, in a disease where medical assistance is never crowned with success. The extirpation of the cancerous mammæ, so frequently executed with success, guided my ideas on the analogical extirpation of the uterus, an organ which is known to be in close connexion with the mammæ, that such an analogical conclusion might be excusable, even if our experience did not inform us of the possibility of such an operation. Not only in old but also in modern writers, we find cases of this operation noticed, of which, particularly, I shall only mention a case described by Professor Wrisberg, (Commentatio de uteri mox post partum naturalem resectione non lethale, &c. Gottingæ, 1787), in which the uterus was cut off by a rude ignorant midwife, who took the prolapsus uteri for a preternatural excrescence. The wound was afterwards healed with only the assistance of nature; and the person, whom I myself had an opportunity of examining twenty years after that accident, enjoyed tolerably good health. This case, however, seems to prove how little danger this operation is attended with, which I think could be safely undertaken, in case no adhesions of the diseased uterus to the neighbouring parts take place, which some physicians think to be always the case in cancer uteri, but which, however, is discredited by others.

The operation cannot be performed unless a prolapsus uteri is previously occasioned; and if it should not exist together with the cancer, as I have found it in one case, I think it a very essential point of the operation, to produce it by art, which is generally done without much difficulty, as the ligaments of the uterus are in most cases very much relaxed. But to make this operation more easy, I should advise to prepare the uterus, during some weeks, by emollient injections with opium; and the parts being thus sufficiently relaxed, the attempt must be made for making a prolapsus uteri, as the first step in the whole operation. For this purpose I recommend the instrument A. *(see the plate)* which is made of good steel and *one* foot long; the branches are round and well polished, being shut in the manner of a pair of midwifery forceps; the upper

 end end of each branch is provided with a knob surrounded with soft pads of leather; the handle is like that of a pair of scissars; the upper part is gently curved outwards, so as to leave a space of about one inch and a quarter, when the handle is shut.

In order to produce the prolapsus, the patient should be placed on a table covered with beds, so as to rest on the knees and the elbows, and the thighs must be drawn from one another to be able to introduce the instrument, which the operator is to do in the same way as when he brings a forceps through the orifice of the uterus. The instrument being shut, he is to put the two indices into the ring *x*. depressing the thumbs towards *u*, and the middle fingers to *o*, and thus to keep the instrument shut. The operator is to make every day about twenty tractions, injecting from time to time an emollient decoction with opium, till he has gradually drawn down the uterus, while he endeavours to keep the vagina in its proper situation. When the prolapsus is in this manner effected, I recommend the curved knife B, to separate with a circular cut the vaginal portion and the neck of the uterus. The rest of the uterus should now be gradually drawn out with a pair of pincers, to tie the vessels of the vagina, and the remaining portion must be kept outwards, partly for preventing any blood getting into the abdomen, partly for applying the dressing proper for healing the wound.

## Figures and Tables

**Figure f1:**